# The association between hypoxia inducible factor 1 subunit alpha gene rs2057482 polymorphism and cancer risk: a meta-analysis

**DOI:** 10.1186/s12885-019-6329-2

**Published:** 2019-11-19

**Authors:** Li-Fang Wu, Gui-Ping Xu, Qing Zhao, Li-Jing Zhou, Ding Wang, Wei-Xian Chen

**Affiliations:** 1 Department of Laboratory Medicine, The Second Affiliated Hospital of Chongqing Medical University, Chongqing, 400010 China; 2 Transfusion Department, The Second Affiliated Hospital of Chongqing Medical University, Chongqing, China

**Keywords:** *HIF1A*, Cancer, Meta-analysis, Polymorphism, rs2057482

## Abstract

**Background:**

The rs2057482 polymorphism in the *hypoxia inducible factor 1 subunit alpha (HIF1A)* gene has been reported to be associated with a risk of several types of cancer, but this association has not yet been definitively confirmed. We performed this meta-analysis to determine whether rs2057482 is associated with overall cancer risk.

**Methods:**

The PubMed, Embase, and Web of Science databases were searched for the potential studies about the association between the rs2057482 and cancer risk. The data of genotype frequencies in cases with cancer and controls were extracted from the selected studies. Odds ratios (ORs) and the corresponding 95% confidence intervals (CIs) were calculated to determine the strength of the associations.

**Results:**

The meta-analysis showed an association between the rs2057482 polymorphism and overall cancer risk. However, a stratified analysis of ethnicity did not show any significant association between rs2057482 and cancer risk in the Asian population.

**Conclusions:**

The rs2057482 polymorphism was associated with decreased overall cancer risk, based on the currently available studies. However, this conclusion needs verification by further well-designed epidemiology studies that examine different cancer types and more subjects.

## Background

Hypoxia inducible factor 1 (HIF1) is a transcription factor that coordinates the response to hypoxia in cells and tissues [1]. HIF1 is a heterodimeric protein consisting of HIF1A and HIF1B subunits. The HIF1A subunit is induced by hypoxia and forms an active transcription inducer with HIF1B [2]. *HIF1* activation promotes the expression of several hypoxia-adaptation genes, including those involved in the biological processes of angiogenesis, erythropoiesis, cell proliferation, and apoptosis [3, 4].

Hypoxia is a common issue in tumor tissues [5]. HIF1A regulates cellular adaptation to hypoxia and promotes tumor development [6–8]. HIF1A expression increases in various types of cancer, such as breast, prostate, and colon cancer [9], and a high expression of HIF1A in a tumor indicates poor patient prognosis [10]. The relapse-free survival time is significantly lower for the hepatocellular carcinoma patients with high expression of HIF1A than for patients with low expression [11].

The rs2057482 polymorphism is located in the 3′ untranslated region (3′-UTR) region of the *HIF1A* gene. The SNP has been widely explored for its relationship with cancer risk [12–18]. We performed this meta-analysis to provide a more accurate assessment and obtain a comprehensive understanding of the relationship between rs2057482 and cancer risk.

## Methods

### Search strategy

Three databases (PubMed, Embase, and Web of Science) were retrieved up to September 24, 2019, using the following keywords: “*hypoxia inducible factor 1 subunit alpha* or *HIF1A*,” “polymorphism or variant or mutation or SNP,” and “cancer or carcinoma or tumor.” We also checked the Ensembl web site for potential studies (http://asia.ensembl.org/Homo_sapiens/Variation).

### Inclusion and exclusion criteria

The inclusion criteria were:

1. The study is about the relationship between rs2057482 and cancer risk.
2. The study is a case-control study or cohort study.
3. The study must contain sufficient genotype data for the meta-analysis.
4. The study is published in English.

We excluded reviews, meta-analyses, and abstracts.

### Data extraction

The data from the selected studies were extracted by two authors separately. The extracted data were the following: first author; publication year; country or region where the study conducted; control source; genotype methods; and genotype frequencies.

### Quality score

We evaluated the quality of these included studies by scoring each study based on the case and control source, number of subjects, and Hardy-Weinberg equilibrium (Additional file 1: Table S1) [19].

### Statistical analysis

Statistical analyses were carried out using the Stata software (Version 12.0, Stata Corporation, College Station, TX). ORs and 95%Cls were calculated to evaluate the strength of the association between the rs2057482 polymorphism and cancer risk. *P* values < 0.05 were considered statistically significant. This meta-analysis used five genetic models: the allele (T vs. C), homozygote (TT vs. CC), heterozygote (CT vs. CC), dominant (TT + CT vs. CC), and recessive models (TT vs. CT + CC). We also conducted stratified analysis of ethnicity; however, only one study was about the Caucasian population, so we have only shown results for the Asian population. In addition, only one study was retrieved about each type of cancer, so we did not conduct a stratified analysis of cancer type. We measured the heterogeneity with the parameter I^2^ and the *P*-value with the Chi-squared test [20]. When I^2^ < 50% or *P* > 0.10, the fixed model was used [21]; otherwise, a random model was used [22]. Sensitivity analyses were performed by removing one study each time [23]. Publication bias was determined using the Egger and Begg tests [24, 25].

## Results

### Characteristics of the studies

**Fig. 1:**
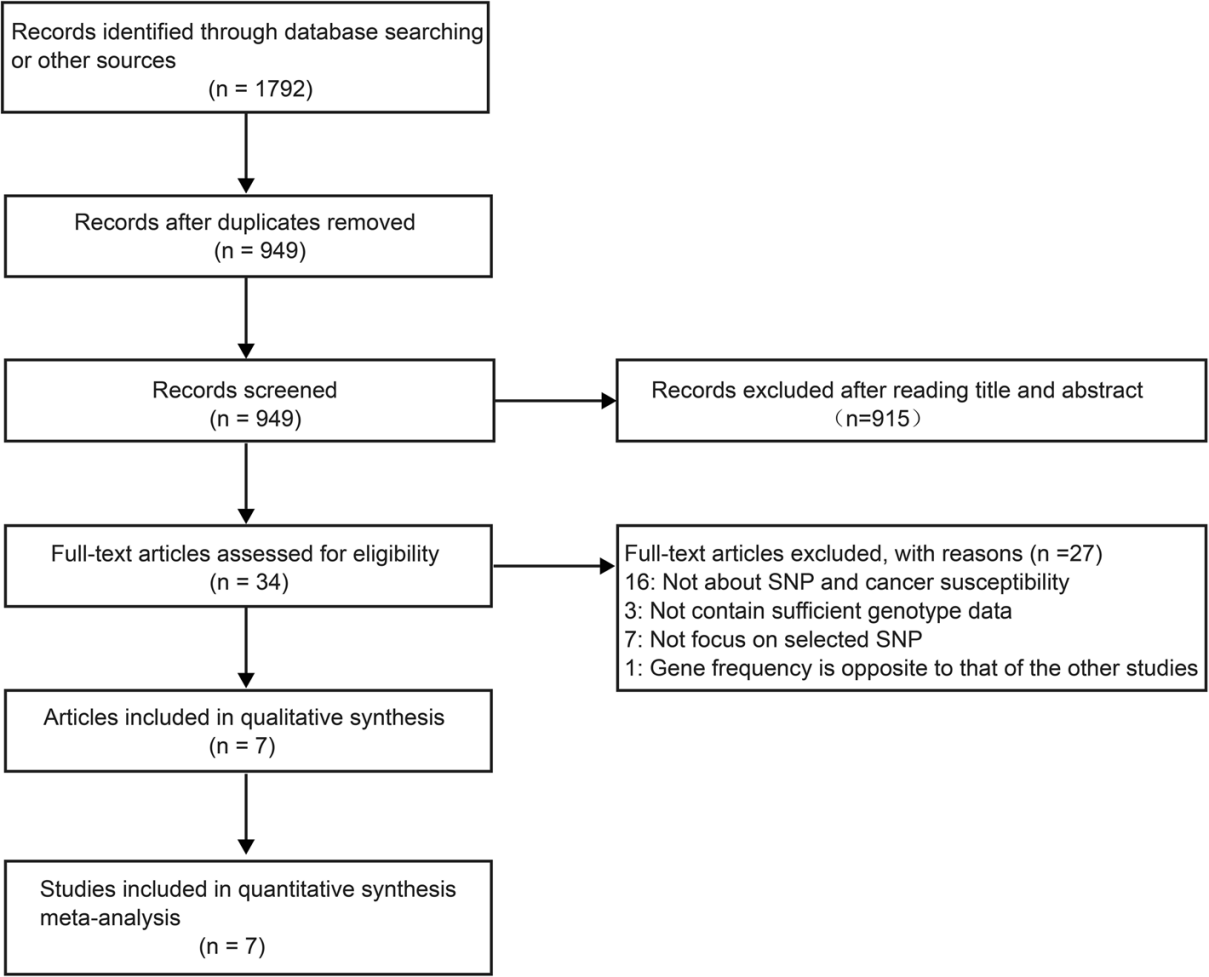
The flow diagram of included/excluded studies

The process used to select target articles is shown in Fig. 1. The database searches identified 949 studies after removing duplicate records. A further check of the titles and abstracts excluded 915 studies. We read the full text of the remaining 34 studies and ultimately selected 7 studies for the meta-analysis. The included studies were conducted between 2008 and 2018; five studies were about the Asian population, one was about the Caucasian population, and one was about a mixed population. The characteristics of these studies are shown in Table 1. The genotype frequencies are listed in Table 2.

**Table 1:** Characteristics of the studies included in the meta-analysis

First author	Year	Country/ Region	Ethnicity	Cancer type	Genotyping method	Control source
Lee (12)	2008	Korea	Asian	breast cancer	SNP-IT™ assays	PB
Qin (13)	2011	China	Asian	RCC	Taqman	HB
Li (14)	2012	China	Asian	prostate cancer	Taqman	HB
Wang (15)	2016	China	Asian	PDAC	DNA sequence	PB
Yamamoto (16)	2016	Japan	Asian	lung cancer	Taqman	HB
Gregory (17)	2016	USA	Mix	NHL	Fluidigm Dynamic 96.96 Array™ assay	HB
Martina (18)	2018	Czech	Caucasian	MM	Taqman	HB

RCC, renal cell carcinoma; PDAC, pancreatic ductal adenocarcinoma; NHL: non-Hodgkin lymphoma; MM: multiple myeloma; PB, population-based; HB, hospital-based

**Table 2:** *HIF1A* rs2057482 polymorphism genotype distribution and allele frequency in cases and controls

	Genotype(N)								Allele frequency (N)				HWE	Score
	Case				Control				Case		Control			
	Total	CC	CT	TT	Total	CC	CT	TT	C	T	C	T		
Lee 2008 (12)	1150	691	415	44	1048	611	396	41	1797	503	1618	478	0.018	11
Qin 2011 (13)	620	388	196	36	623	393	201	29	972	268	987	259	0.613	12
Li 2012 (14)	662	418	212	32	716	428	241	47	1048	276	1097	335	0.103	12
Wang 2016 (15)	410	301	69	40	490	302	154	34	671	149	758	222	0.022	10
Yamamoto 2016 (16)	462	302	138	22	379	244	121	14	742	182	609	149	0.834	11
Gregory 2016 (17)	180	125	49	6	528	369	147	12	299	61	885	171	0.554	11
Martina 2018 (18)	275	225	47	3	219	176	39	4	497	53	391	47	0.297	10

HWE: Hardy-Weinberg equilibrium

### Meta-analysis

**Fig. 2:**
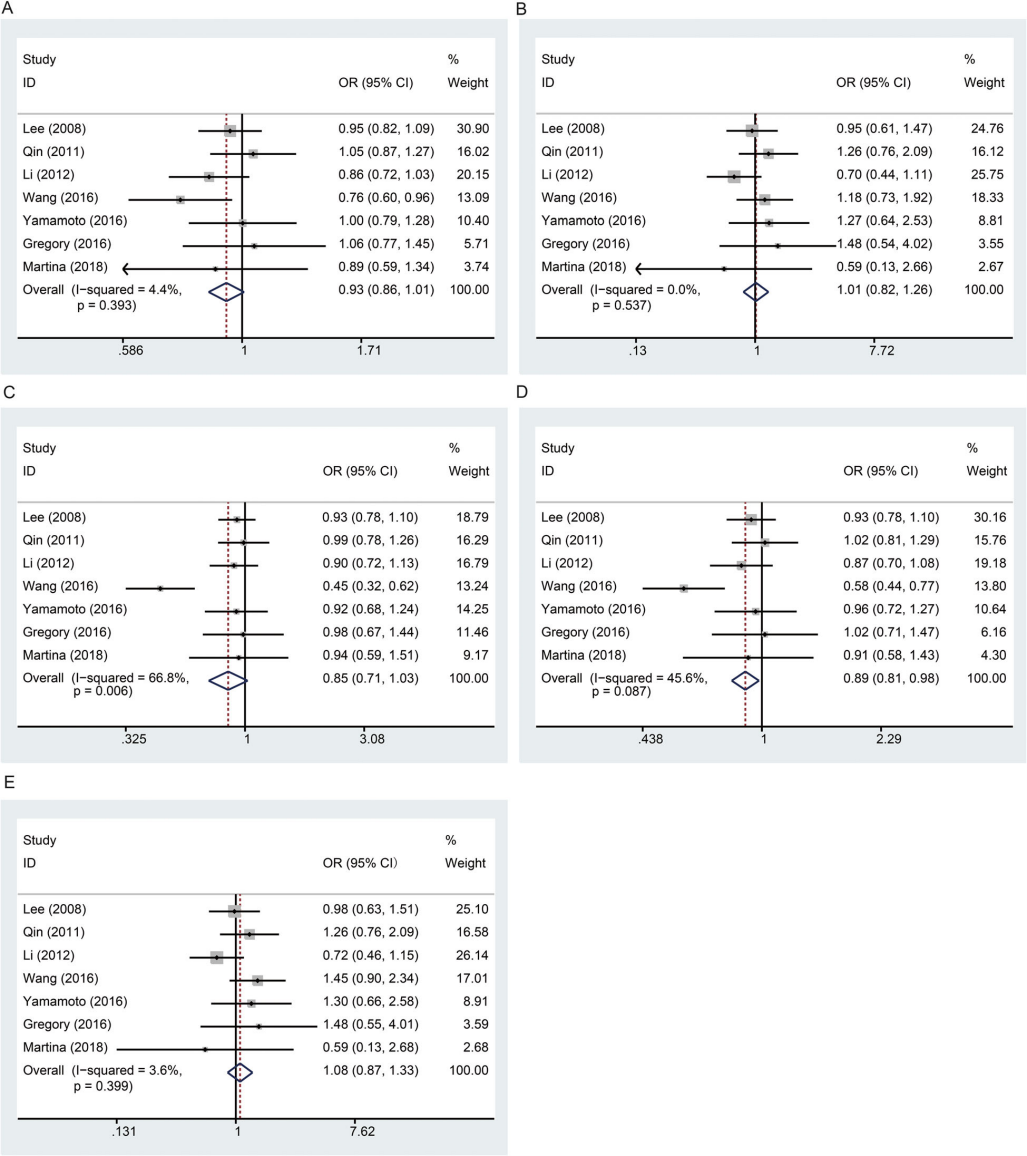
Meta-analysis of the association between rs2057482 and risk of cancer **a**: allele model; **b**: homozygous model; **c**: heterozygous model; D: dominant model; E: recessive model**.** The squares and horizontal lines correspond to the study specific OR and 95% CI. The area of the squares reflects the weight. The diamond represents the summary OR and 95% CI. The random-effects model was used for the heterozygous genetic model, and fixed-effects models were used for other genetic models.

In this meta-analysis, the overall analysis under the dominant genetic model showed a significant association between the rs2057482 polymorphism and a decreased risk of cancer (Table 3 and Fig. 2, TT + CT vs. CC: OR, 0.89, 95% CI, 0.81–0.98, *P* = 0.017). However, the stratified analysis of ethnicity did not indicate an association between rs2057482 and cancer risk in the Asian population in any of the genetic models (Table 3). We only synthesized the results if two or more studies were available, so the results for the Caucasian population were not shown.

**Table 3:** Meta-analysis of the association between rs2057482 polymorphism and cancer susceptibility

Subgroup	No.	T vs. C			TT vs. CC			CT vs. CC			TT + CT vs. CC			TT vs. CT + CC		
		OR(95%Cl)	*P*_*OR*_	I^2^	OR(95%Cl)	*P*_*OR*_	I^2^	OR(95%Cl)	*P*_*OR*_	I^2^	OR(95%Cl)	*P*_*OR*_	I^2^	OR(95%Cl)	*P*_*OR*_	I^2^
Overall	7	0.93(0.86–1.01)	0.081	4.4%	1.01(0.82–1.26)	0.903	0.0%	0.85(0.71–1.03)^*^	0.091	66.8%	**0.89(0.81–0.98)**	**0.017**	45.6%	1.08(0.87–1.33)	0.502	3.6%
Asian	5	0.93(0.85–1.01)	0.070	28.8%	1.01(0.81–1.26)	0.943	0.2%	0.82(0.65–1.04)^*^	0.096	77.2%	0.87(0.73–1.03)^*^	0.095	61.8%	1.08(0.86–1.34)	0.526	23.4%

OR*,* odds ratio; 95% CI, 95% confidence interval; *P*_*OR*_, pool *P* value; RCC, renal cell carcinoma; *indicates that the OR, 95% Cl, and corresponding *P*_*OR*_ were calculated based on the random-effects model; otherwise, the fixed-effects model was used. Bold values are statistically significant (P_OR_ < 0.05)

### Sensitivity analysis

**Fig. 3:**
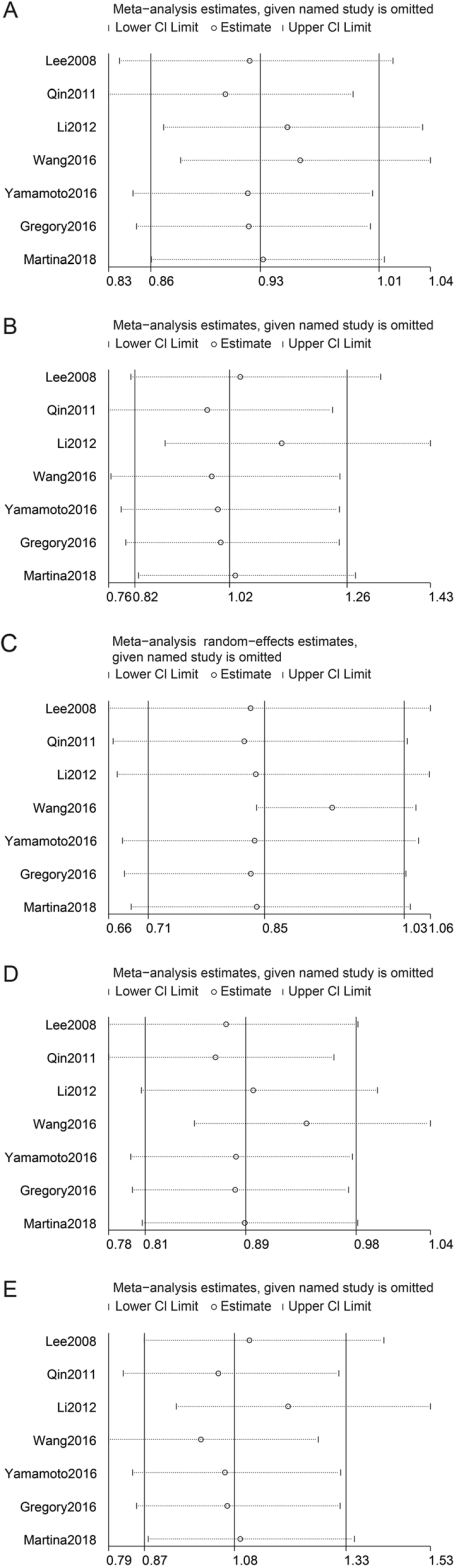
Sensitivity analyses between rs2057482 polymorphism and risk of cancer **a**: allele model; **b**: homozygous model; **c**: heterozygous model; **d**: dominant model; **e**: recessive model**.** The random-effects model was used for the heterozygous genetic model, and fixed-effects models were used for other genetic models.

Sensitivity analyses were performed using the *metainf* command. Exclusion of the Qin2011 study led to a different result in the allele model, and exclusion of the Wang2016 study led to a different result in the dominant model (Fig. 3 and Additional file 1: Table S2). We also preformed sensitivity analyses in Asian populations. Exclusion of the Qin2011 study led to a different result in the allele model (Additional file 1: Table S3). These results suggest that our results were not stable in these models.

### Publication bias

Egger and Begg tests carried out to detect potential publication bias revealed no publication bias in this meta-analysis (Table 4).

**Table 4:** Publication bias analysis

Genetic model	Egger’s test			Begg’s test
	t	95% Cl	*P*	*P*
T vs. C	−0.03	−3.644~3.573	0.981	1.000
TT vs. CC	0.23	−2.671~3.207	0.824	0.764
CT vs. CC	−0.60	−7.483~4.660	0.576	0.548
TT + CT vs. CC	−0.28	−5.559~4.470	0.791	0.764
TT vs. CT + CC	0.09	−3.138~3.371	0.930	1.000

## Discussion

HIF1A plays a central role in tumor adaptation to hypoxia [26, 27]. HIF1A mediates the hypoxic adaptation of tumor cells and tissues in multiple ways [28]. For example, angiogenesis is an important aspect of cancer progression, and HIF1A contributes to tumor angiogenesis by upregulating vascular endothelial growth factor (VEGF) and other proangiogenic factors [29, 30]. HIF1A also increases the expression of multiple enzymes involved in glycolysis, which further aids tumor cell growth and proliferation [31–34]. In addition, HIF1A promotes autophagy by altering the expression of BCL2/adenovirus E1B 19 kDa protein-interacting protein 3 (BNIP3), which is part of a stress adaptation mechanism that promotes tumor cell survival and avoids cell death [35, 36]. Many studies have reported associations between *HIF1A* polymorphisms and the risks of various types of cancer, including bladder, oral, and colorectal cancers, head and neck squamous cell carcinoma, and renal cell carcinoma [37–40].

The *HIF1A* gene has many SNPs, but we focused on rs2057482 for the following reasons: First, the relationship between rs2057482 and cancer risk has been reported in previous epidemiology studies [12–18]. Second, in the 1000 Genomes Project Phase 3, minor allele frequencies of rs2057482 are greater than 10% in most populations (Additional file 1: Table S4). Third, the selected SNP may have important biological functions, according to previous reports.

The rs2057482 polymorphism is located in the 3’UTR of *HIF1A*. Many researchers have hypothesized that this polymorphism may be located near the microRNA binding site and that it affects the expression of HIF1A by binding HIF1A to the microRNA [13, 15–18]. Wang et al. reported that rs2057482 may affect the expression of HIF1A by microRNA 199a [15]. Gregory et al., based on silico analyses with the miRNA-SNP analytic tool TargetScan 5.2, suggested that the T allele of *HIF1A* rs2057482 created new microRNA binding sites for microRNA 196a-2 [17].

The rs2057482 polymorphism has been reported to associate with the occurrence and prognosis of several kinds of cancer [41–44]. For example, this polymorphism was found to decrease the risk of non-Hodgkin’s lymphoma associated with central nervous system acquired immune deficiency syndrome [17]. An association has also been reported between rs2057482 polymorphism and prognosis of early-stage lung cancer patients after surgery [45]. In addition, the recurrence rate of hepatocellular carcinoma is lower in CT + TT carrier patients than in CC carrier patients [11].

Our meta-analysis revealed that rs2057482 decreased the overall cancer risk in the dominant genetic model. We hypothesize that the carrier of the T allele (CT + TT) creates a new microRNA binding site. Binding leads to decreased expression of HIF1A, thereby reducing the risk of cancer. However, this conclusion is made based on only seven types of tumor (breast, lung, prostate cancer, pancreatic ductal adenocarcinoma, renal cell carcinoma, non-Hodgkin’s lymphoma, and multiple myeloma); therefore, we suggest that the relationship between rs2057482 and more types of cancer should be investigated in further studies.

The current meta-analysis has the following limitations that should be recognized. First, the number of studies contained in the meta-analysis is limited, and the majority of the study populations were Asians. The genomic effect of this SNP could be ethnicity specific, so this prevalence of Asian subjects may have biased the results for overall cancer risk. The present study results therefore only provide meaningful information for Asian populations, and we recommend additional research on the risk of cancer and rs2057482 in Caucasian and other populations in the future. Second, only one study was included for each cancer type, so we did not conduct a stratified analysis based on cancer type. Each type of cancer may have a different underlying genomic mechanism, so more studies on rs2057482 and cancer risk are needed for each type of cancer. Finally, the mechanism by which this SNP affects tumor risk is still unclear and needs further exploration.

## Conclusions

Our meta-analysis suggests that the rs205782 polymorphism of the *HIF1A* gene significantly decreases the overall cancer risk, based on the synthesis results of the included studies. This conclusion should be further verified by additional studies that include more subjects and cancer types.

## Supplementary information

**Additional file 1: Table S1.** Quality score assessment. **Table S2.** Sensitivity analyses for rs2057482 polymorphism and cancer susceptibility. **Table S3.** Sensitivity analyses for rs2057482 polymorphism and cancer susceptibility in Asian population. **Table S4.** MAFs of rs2057482 polymorphism in the populations from the 1000 Genomes Project Phase 3.

## Data Availability

All data generated or analysed during this study are included in this published article and its supplementary information files.

## Abbreviation

3′-UTR: three prime untranslated region
BNIP3: BCL2/adenovirus E1B 19 kDa protein-interacting protein 3
CI: confidence interval
HB: hospital-based
HIF1: hypoxia inducible factor 1
HWE: Hardy-Weinberg equilibrium
MAF: minor allele frequency
MM: multiple myeloma
NHL: non-Hodgkin lymphoma
OR: odds ratio
PB: population-based
PDAC: pancreatic ductal adenocarcinoma
RCC: renal cell carcinoma
SNP: single nucleotide polymorphism
VEGF: vascular endothelial growth factor
