# Supplementary material for: The association between hypoxia inducible factor 1 subunit alpha gene rs2057482 polymorphism and cancer risk: a meta-analysis
Source: BMC Cancer. 2019 Nov 19;19:1123. doi: 10.1186/s12885-019-6329-2 (PMC6862742; doi:10.1186/s12885-019-6329-2)
Supplement: Supplementary file 1 — Additional file 1: Table S1. Quality score assessment. Table S2. Sensitivity analyses for rs2057482 polymorphism and cancer susceptibility. Table S3. Sensitivity analyses for rs2057482 polymorphism and cancer susceptibility in Asian population. Table S4. MAFs of rs2057482 polymorphism in the populations from the 1000 Genomes Project Phase 3. [file 12885_2019_6329_MOESM1_ESM.docx]

**Scale for quality assessment criterion (19)**

|  | Criterion | Score |
| --- | --- | --- |
| **A** | **Source of cases** |  |
|  | Selected from population or cancer registry | 3 |
|  | Selected from hospital | 2 |
|  | Selected from pathology archives, but without description | 1 |
|  | Not described | 0 |
| **B** | **Source of controls** |  |
|  | Population-based | 3 |
|  | Blood donors or volunteers | 2 |
|  | Hospital-based (cancer-free patients) | 1 |
|  | Not described | 0 |
| **C** | **Specimens used for determining genotypes** |  |
|  | White blood cells or normal tissues | 3 |
|  | Not mentioned | 2 |
|  | Tumor tissues or exfoliated cells of tissue | 0 |
| **D** | **Hardy–Weinberg equilibrium in controls** |  |
|  | Hardy–Weinberg equilibrium | 3 |
|  | Hardy–Weinberg disequilibrium | 0 |
| **E** | **Total sample size** |  |
|  | ≥1,000 | 3 |
|  | ≥500 and <1,000 | 2 |
|  | ≥200 and <500 | 1 |
|  | <200 | 0 |

**Table S1: Quality score assessment**

|  | **A** | **B** | **C** | **D** | **E** | **Score** |
| --- | --- | --- | --- | --- | --- | --- |
| Lee 2008(12) | 2 | 3 | 3 | 0 | 3 | 11 |
| Qin 2011 (13) | 2 | 1 | 3 | 3 | 3 | 12 |
| Li 2012 (14) | 2 | 1 | 3 | 3 | 3 | 12 |
| Wang 2016(15) | 2 | 3 | 3 | 0 | 2 | 10 |
| Yamamoto 2016 (16) | 2 | 1 | 3 | 3 | 2 | 11 |
| Gregory 2016 (17) | 2 | 1 | 3 | 3 | 2 | 11 |
| Martina 2018 (18) | 2 | 1 | 3 | 3 | 1 | 10 |

A-E represents the corresponding criterion in the table of scale for quality assessment criterion.

**Table S2: Sensitivity analyses for rs2057482 polymorphism and cancer susceptibility**

| **Comparison** | **Study omitted** | **Estimate** | **[95% Confident Interval]** | **Effect model** |
| --- | --- | --- | --- | --- |
| **T vs. C** | Lee 2008 | 0.925 | 0.841-1.018 | Fix |
|  | **Qin 2011** | **0.909** | **0.834-0.992** |  |
|  | Li 2012 | 0.950 | 0.869-1.038 |  |
|  | Wang 2016 | 0.958 | 0.880-1.042 |  |
|  | Yamamoto 2016 | 0.924 | 0.849-1.005 |  |
|  | Gregory 2016 | 0.925 | 0.852-1.004 |  |
|  | Martina 2018 | 0.934 | 0.861-1.009 |  |
|  | Combined | 0.932 | 0.861-1.009 |  |
| **TT vs. CC** | Lee 2008 | 1.038 | 0.810-1.331 | Fix |
|  | Qin 2011 | 0.969 | 0.763-1.231 |  |
|  | Li 2012 | 1.124 | 0.881-1.435 |  |
|  | Wang 2016 | 0.978 | 0.768-1.246 |  |
|  | Yamamoto 2016 | 0.991 | 0.790-1.245 |  |
|  | Gregory 2016 | 0.997 | 0.799-1.245 |  |
|  | Martina 2018 | 1.028 | 0.826-1.278 |  |
|  | Combined | 1.016 | 0.818-1.261 |  |
| **CT vs. CC** | Lee 2008 | 0.836 | 0.661-1.057 | Random |
|  | Qin 2011 | 0.828 | 0.667-1.029 |  |
|  | Li 2012 | 0.842 | 0.672-1.056 |  |
|  | Wang 2016 | 0.937 | 0.844-1.040 |  |
|  | Yamamoto 2016 | 0.841 | 0.678-1.043 |  |
|  | Gregory 2016 | 0.836 | 0.681-1.027 |  |
|  | Martina 2018 | 0.844 | 0.689-1.033 |  |
|  | Combined | 0.853 | 0.710-1.025 |  |
| **TT+CT vs. CC** | Lee 2008 | 0.877 | 0.782-0.983 | Fix |
|  | Qin 2011 | 0.868 | 0.783-0.963 |  |
|  | Li 2012 | 0.899 | 0.809-0.998 |  |
|  | **Wang 2016** | **0.941** | **0.851-1.041** |  |
|  | Yamamoto 2016 | 0.885 | 0.800-0.978 |  |
|  | Gregory 2016 | 0.884 | 0.802-0.975 |  |
|  | Martina 2018 | 0.892 | 0.809-0.982 |  |
|  | Combined | 0.893 | 0.812-0.981 |  |
| **TT vs. CT+CC** | Lee 2008 | 1.112 | 0.870-1.422 | Fix |
|  | Qin 2011 | 1.041 | 0.822-1.318 |  |
|  | Li 2012 | 1.201 | 0.944-1.529 |  |
|  | Wang 2016 | 1.001 | 0.788-1.271 |  |
|  | Yamamoto 2016 | 1.056 | 0.844-1.322 |  |
|  | Gregory 2016 | 1.062 | 0.853-1.321 |  |
|  | Martina 2018 | 1.091 | 0.880-1.354 |  |
|  | Combined | 1.078 | 0.871-1.335 |  |

**Table S3: Sensitivity analyses for rs2057482 polymorphism and cancer susceptibility in Asian population**

| **Comparison** | **Study omitted** | **Estimate** | **[95% Confident Interval]** | **Effect model** |
| --- | --- | --- | --- | --- |
| **T vs. C** | Lee 2008 | 0.915 | 0.825-1.015 | Fix |
|  | **Qin 2011** | **0.899** | **0.819-0.987** |  |
|  | Li 2012 | 0.944 | 0.859-1.038 |  |
|  | Wang 2016 | 0.954 | 0.872-1.044 |  |
|  | Yamamoto 2016 | 0.916 | 0.838-1.002 |  |
|  | Combined | 0.926 | 0.852-1.007 |  |
| **TT vs. CC** | Lee 2008 | 1.031 | 0.795-1.337 | Fix |
|  | Qin 2011 | 0.957 | 0.746-1.228 |  |
|  | Li 2012 | 1.125 | 0.872-1.452 |  |
|  | Wang 2016 | 0.967 | 0.751-1.245 |  |
|  | Yamamoto 2016 | 0.982 | 0.775-1.244 |  |
|  | Combined | 1.009 | 0.807-1.262 |  |
| **CT vs. CC** | Lee 2008 | 0.790 | 0.575-1.084 | Random |
|  | Qin 2011 | 0.782 | 0.587-1.041 |  |
|  | Li 2012 | 0.798 | 0.590-1.081 |  |
|  | Wang 2016 | 0.932 | 0.834-1.042 |  |
|  | Yamamoto 2016 | 0.798 | 0.602-1.059 |  |
|  | Combined | 0.823 | 0.654-1.035 |  |
| **TT+CT vs. CC** | Lee 2008 | 0.845 | 0.672-1.063 | Random |
|  | Qin 2011 | 0.830 | 0.682-1.011 |  |
|  | Li 2012 | 0.863 | 0.692-1.076 |  |
|  | Wang 2016 | 0.937 | 0.841-1.043 |  |
|  | Yamamoto 2016 | 0.847 | 0.691-1.037 |  |
|  | Combined | 0.867 | 0.733-1.025 |  |
| **TT vs. CT+CC** | Lee 2008 | 1.111 | 0.860-1.437 | Fix |
|  | Qin 2011 | 1.034 | 0.809-1.323 |  |
|  | Li 2012 | 1.209 | 0.940-1.555 |  |
|  | Wang 2016 | 0.991 | 0.772-1.271 |  |
|  | Yamamoto 2016 | 1.051 | 0.832-1.327 |  |
|  | Combined | 1.075 | 0.862-1.341 |  |

**Table S4: MAFs of rs2057482 polymorphism in the populations from the 1000 Genomes Project Phase 3**

| Populations | MAF |
| --- | --- |
| ACB | 0.339 |
| ASW | 0.328 |
| BEB | 0.267 |
| CDX | 0.199 |
| CEU | 0.101 |
| CHB | 0.209 |
| CHS | 0.152 |
| CLM | 0.170 |
| ESN | 0.374 |
| FIN | 0.101 |
| GBR | 0.143 |
| GIH | 0.199 |
| GWD | 0.350 |
| IBS | 0.131 |
| ITU | 0.373 |
| JPT | 0.216 |
| KHV | 0.222 |
| LWK | 0.475 |
| MSL | 0.306 |
| MXL | 0.133 |
| PEL | 0.071 |
| PJL | 0.297 |
| PUR | 0.231 |
| STU | 0.319 |
| TSI | 0.229 |
| YRI | 0.333 |

MAFs: minor allele frequencies; ACB: African Carribbeans in Barbados; ASW: Americans of African Ancestry in SW USA; BEB: Bengali from Bangladesh; CDX: Chinese Dai in Xi -shuangbanna, China; CEU: Utah Residents with Northern and Western European Ancestry; CHB: Han Chinese in Beijing, China; CHS: Southern Han Chinese; CLM: Colombians from Medellin, Colombia; ESN: Esan in Nigeria; FIN: Finnish in Finland; GBR: British in England and Scotland; GIH: Gujarati Indian from Houston, Texas; GWD: Gambian in Western Division in the Gambia; IBS: Iberian Population in Spain; ITU: Indian Telugu from the UK; JPT: Japanese in Tokyo, Japan; KHV: Kinh in Ho Chi Minh City, Vietnam; LWK: Luhya in Webuye, Kenya; MSL: Mende in Sierra Leone; MXL: Mexican Ancestry in Los Angeles USA; PEL: Peruvians from Lima, Peru; PJL:  Punjabi from Lahore, Pakistan; PUR: Puerto Rican in Puerto Rico; STU: Sri Lankan Tamil from the UK; TSI: Toscani in Italia; YRI: Yoruba in Ibadan, Nigeria; N/A: MAF not available in these populations in 1000 Genomes.
